# Endothelial Activation and Stress Index (EASIX) as an Early Predictor for Mortality and Overall Survival in Hematological and Non-Hematological Patients with COVID-19: Multicenter Cohort Study

**DOI:** 10.3390/jcm10194373

**Published:** 2021-09-24

**Authors:** Elżbieta Kalicińska, Monika Biernat, Justyna Rybka, Aleksander Zińczuk, Justyna Janocha-Litwin, Marta Rosiek-Biegus, Marta Morawska, Anna Waszczuk-Gajda, Joanna Drozd-Sokołowska, Łukasz Szukalski, Marcin Rymko, Paula Jabłonowska, Krzysztof Simon, Tomasz Wróbel

**Affiliations:** 1 Department and Clinic of Hematology, Blood Neoplasms, and Bone Marrow Transplantation, Wrocław Medical University, 50-367 Wrocław, Poland; mobiernat@gmail.com (M.B.); rybka.justyna@o2.pl (J.R.); pjablonowska@usk.wroc.pl (P.J.); tomasz_wrobel@wp.pl (T.W.); 2 Department of Infectious Diseases and Hepatology, Wrocław Medical University, 51-149 Wrocław, Poland; alek.zinczuk@gmail.com (A.Z.); justynajanocha@o2.pl (J.J.-L.); krzysztof.simon@umed.wroc.pl (K.S.); 3 Department of Forensic Medicine, Wrocław Medical University, 50-372 Wrocław, Poland; 4 Department of Internal Medicine, Pneumology and Allergology, Wrocław Medical University, 50-367 Wrocław, Poland; martarosiek@gmail.com; 5 Experimental Hematooncology Department, Medical University of Lublin, 20-093 Lublin, Poland; mmorawska79@gmail.com; 6 Hematology Department, St. John’s Cancer Center, 20-090 Lublin, Poland; 7 Department of Hematology, Transplantation and Internal Medicine, Medical University of Warsaw, 02-097 Warsaw, Poland; annawaszczukgajda@gmail.com (A.W.-G.); johna.dr@poczta.fm (J.D.-S.); 8 Department of Hematology, Collegium Medicum in Bydgoszcz, Nicolaus Copernicus University in Torun, 85-168 Bydgoszcz, Poland; lukaszszukalski@gmail.com; 9 Department of Hematology and Bone Marrow Transplantation, SSM Nicolaus Copernicus, 87-100 Toruń, Poland; rymkom@gmail.com

**Keywords:** endothelial dysfunction, EASIX, COVID-19, hematological cancer, lactate dehydrogenase, platelet count, creatinine

## Abstract

COVID-19, as a disease involving the endothelium of multiple organs, is characterized by high mortality rates among hospitalized patients. Patients with hematological malignancies are particularly at risk of an unfavorable course of COVID-19. The endothelial activation and stress index (EASIX) score has been used as a simple predictor of overall survival (OS) in specific groups of hematological cancer patients. EASIX, as a biomarker of endothelial dysfunction, might play a prognostic role in patients with COVID-19. Here, we performed a comprehensive retrospective analysis of the EASIX score in 523 hospitalized COVID-19 patients with or without coexisting hematological cancer. Hematological cancer COVID-19 patients had higher EASIX scores compared to the overall population with COVID-19. In hematological patients, EASIX was a strong predictor of the occurrence of sepsis during COVID-19. Our findings demonstrated EASIX as a strong predictor of intensive care unit admission, in-hospital mortality, the occurrence of acute renal failure and the need for hemodialysis, both in hematological and non-hematological COVID-19 patients. Patients with a high EASIX score on COVID-19 diagnosis had significantly inferior OS compared to patients with low EASIX. We showed for the first time that EASIX might serve as a simple, universal prognostic tool of OS in both hematological and non-hematological COVID-19 patients.

## 1. Introduction

The novel COVID-19 pandemic has paralyzed many health systems throughout the world and has contributed to significant mortality among the worldwide population. SARS-CoV-2 infection causes severe illness, particularly in certain groups of patients. Patients with hematological cancer are particularly vulnerable to an unfavorable course of COVID-19 and poor outcome [1,2]. Finding a reliable and accurate predictor of the course of the SARS-CoV-2 infection might be beneficial during the early clinical evaluation and treatment of COVID-19 patients as well as in reducing mortality. Prognostic tools for COVID-19 outcome have been investigated recently. According to the meta-analyses, there are multiple risk factors associated with a poor clinical outcome of COVID-19 [3,4]. However, little is known about the establishment of a universal prediction tool for COVID-19 prognosis that is accurate and reliable both in the general and hematological population of COVID-19 patients. The endothelial activation and stress index (EASIX), calculated by the formula LDH (U/L) × Creatinine (mg/dL)/platelet count (10^9^/L), first presented by German and American (US) groups, is a valuable tool to assess the outcome of acute graft-versus-host disease after allogeneic stem cell transplantation, as well as prognosis in patients with lower-risk myelodysplastic syndromes who are not candidates for allogeneic stem cell transplantation [5,6]. Moreover, a Korean group showed that EASIX might also serve as a simple and powerful predictor of survival outcomes in patients with newly diagnosed multiple myeloma [7]. Previous studies in COVID-19 showed that particular components of the EASIX index (platelets, creatinine and LDH), when analyzed separately, might serve as predictors of disease severity [8,9,10,11]. Therefore, it seems reasonable to evaluate the EASIX index in the context of clinical outcome and survival in both hematological and non-hematological COVID-19 patients. 

## 2. Materials and Methods

### 2.1. Study Population

We retrospectively analyzed 523 patients with SARS-CoV-2 infection, hospitalized in 7 Polish medical centers (Department and Clinic of Hematology, Blood Neoplasms and Bone Marrow Transplantation of Wroclaw Medical University, Department of Internal Medicine, Pneumology and Allergology of Wroclaw Medical University, Experimental Hematooncology Department of Medical University in Lublin, Department of Infectious Diseases and Hepatology of Wroclaw Medical University, Department of Hematology of Collegium Medicum in Bydgoszcz, Department of Hematology and Bone Marrow Transplantation SSM Nicolaus Copernicus in Torun, Department of Hematology, Transplantation and Internal Medicine, Medical University of Warsaw) between March 2020 and March 2021, including 125 hematological cancer patients with COVID-19. Criteria defining the clinical status of studied groups of patients were based on COVID-19 treatment guidelines [12]. Asymptomatic COVID-19 infection is defined as a positive SARS-CoV-2 test without symptoms consistent with COVID-19 [12]. Mild illness is defined as the presence of any signs and/or symptoms of COVID-19 but without shortness of breath, dyspnea or abnormal chest imaging [12]. Moderate illness is defined as evidence of lower respiratory disease during clinical assessment or imaging and oxygen saturation of (SpO2) >94% in room air [12]. Severe illness is defined as saturation below 94% in room air, a ratio of arterial partial pressure of oxygen to fraction of inspired oxygen (PaO2/FiO2) <300 mmHg, respiratory frequency >30 breaths/min or lung infiltrates >50% [12]. Critical disease is defined as respiratory failure, septic shock and/or multiple organ dysfunction [12]. The study was performed in accordance with Wroclaw Medical University’s ethics committee (consent no. 315/2020). The collected data did not include any personally identifiable information; therefore, informed consent from the patients was not required. 

### 2.2. Study Design

Patients were divided into 2 groups: (1) those with concomitant hematological cancer, and (2) those without hematological cancer. The hematological malignancies were divided into 5 groups: (1) acute leukemia/MDS EB-2, (2) chronic lymphocytic leukemia/indolent lymphoma, (3) aggressive lymphoma, (4) multiple myeloma and (5) others. After molecular confirmation of SARS-CoV-2 infection (defined by a positive real-time reverse-transcriptase polymerase chain reaction (RT-PCR) assay using nasal and pharyngeal swab specimens), clinical and laboratory data were analyzed. Patient’s characteristics included: age, gender, diagnosis (in case of hematological patients), comorbidities, symptoms of SARS-CoV-2 infection, COVID-19 severity, type of specific COVID-19 treatment, COVID-19 complications, time of hospitalization, basic laboratory parameters, including both hematology and biochemistry indices, and EASIX score calculated using the formula (LDH [U/L] × Creatinine [mg/dl]/platelet count [10^9^/L]. All parameters were analyzed directly after molecular confirmation of SARS-CoV-2 infection. Primary outcome was overall survival (OS), defined as the time from COVID-19 diagnosis to death or last contact. Secondary outcome was in-hospital mortality. Follow-up period was 6 months. 

### 2.3. Statistical Analyses 

A statistical analysis was performed using R version 4.0.5 (The R Foundation for Statistical Computing, Vienna, Austria) and Statistica (version 13.1 software, TIBCO Software Inc., Palo Alto, California, USA) for Windows. Categorical variables were presented as frequencies with percentages, whereas median and interquartile range (IQR) were used to describe continuous variables. Evaluation of data normality was performed using the Shapiro–Wilk test. All continuous variables were non-normally distributed and analyzed using the Mann–Whitney test. Categorical variables were compared using the χ2 test or Fisher’s exact test. The primary endpoint was overall survival rate after 6 months of follow-up, estimated using Kaplan–Meier curves. Log-rank test was applied for intergroup comparison. Hazard ratios and the corresponding 95% confidence intervals were estimated with the use of Cox proportional hazards models. Optimal log2 EASIX cutoff value for survival distribution was determined by maximally selected rank statistics LogRank using exactGauss (log2 EASIX cutoff value = 1.599256, *p* < 0.001) (package “maxstat”, function “maxstat.test”, Erlangen, Germany). EASIX score transformation was validated by calculating the prediction error curve and concordance index curve. Receiver operating characteristics (ROC) curves were used to assess the sensitivity, specificity and area under the ROC curve (AUC) of investigated parameters. Exploratory analyses were performed in hematological and non-hematological COVID-19 subgroups. Analyses were performed using R version 4.0.5 and Statistica 13.1 software for Windows. All statistical tests were two-tailed, with the significance level set at *p* = 0.05.

## 3. Results

### 3.1. Clinical Characteristics of Hematological versus Non-Hematological Patients with COVID-19

The baseline clinical characteristics of the studied subgroups of patients included in the analysis are shown in Table 1. Males comprised 64% of hematological cancer patients with COVID-19 and 51% of COVID-19 patients without hematological malignancy. The median age of patients in the hematological cohort was 62 years, and it was 66 years for patients without a hematological malignancy. Among hematological patients with COVID-19, most were diagnosed with an aggressive malignancy (39% for acute leukemia/myelodysplastic syndrome with excessive blasts-2, and 18% for high-grade lymphomas). The majority of hematological patients were receiving chemotherapy during the SARS-CoV-2 infection (77%). Across the study population, a severe clinical picture of COVID-19 concerned the highest percentage of patients (46%) and was higher in the non-hematological cohort (50% vs. 34%, *p* = 0.003). The median log2 EASIX value was higher in the hematological COVID-19 patients, which indicated significant variation between the studied subgroups (*p* < 0.001). Hematological COVID-19 patients were more often treated with high-flow nasal oxygen and convalescent plasma compared to non-hematological patients (both *p* < 0.001). The median time of hospitalization in the study cohort was 12 days. Mortality rates were higher in the hospitalized hematological cancer patients compared to non-hematological patients with COVID-19 (40% vs. 23%). 

### 3.2. Clinical Characteristics of High EASIX versus Low EASIX COVID-19 Patients

EASIX was evaluated after molecular confirmation of SARS-CoV-2 infection in all studied patients. The optimal EASIX cutoff value for OS was assessed at 1.60 on the log2 scale using maximally selected log-rank statistics (*p* < 0.001). All patients were divided into two groups: 155 patients with high EASIX (log2 EASIX ≥ 1.60), and 367 patients with low EASIX (log2 EASIX < 1.60). Clinical characteristics of high EASIX versus low EASIX COVID-19 patients are shown in Table 2.

Patients with high EASIX were more often males than women (63% vs. 51%, *p* = 0.009) and were characterized by a higher prevalence of hypertension (*p* = 0.003), chronic heart failure (*p* = 0.03) and chronic kidney disease (*p* < 0.001). Among COVID-19 symptoms, dyspnea was more common in patients with a high EASIX (*p* = 0.005). Patients characterized by a high EASIX more often had COVID-19 complications such as DIC (*p* = 0.012), bacterial co-infection, sepsis, acute renal failure, a need for hemodialysis (all *p* < 0.001) and cardiac complications (*p* = 0.001). Patients with a high EASIX also had lower values of all subpopulations of leukocytes (*p* = 0.001), lower values of platelets (*p* < 0.001) and higher levels of D-dimers (*p* < 0.001). High EASIX patients more often required oxygen therapy (*p* = 0.001) and the critical course of COVID-19 was more prevalent in this group (*p* < 0.001).

### 3.3. EASIX and COVID-19 Complications and Outcome

ROC curve analysis was conducted to evaluate the relation among EASIX and COVID-19 complications for the investigated patients, followed by an analysis of the hematological and non-hematological patient subgroups. 

**Figure 1:**
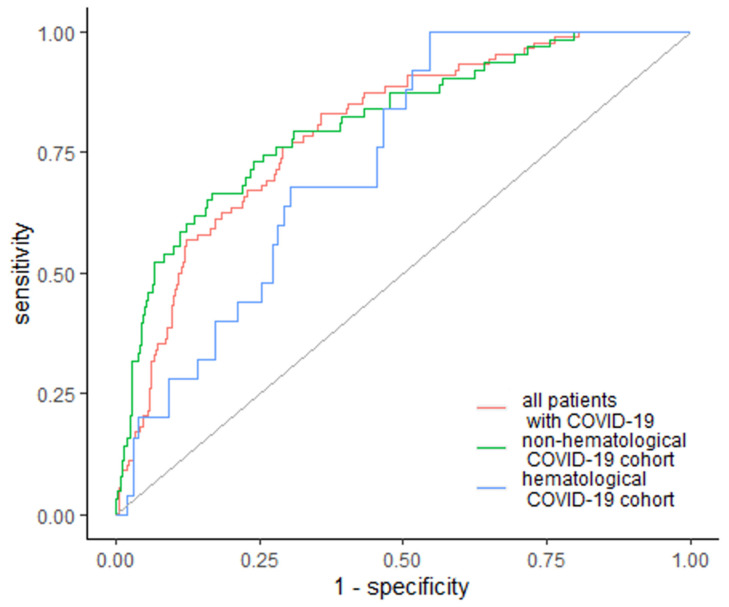
EASIX as a predictor of acute renal failure. Diagonal grey line represents random classifier (false positive rate = true positive rate).

EASIX was the strongest predictor of the occurrence of acute renal failure, including the need for renal dialysis, as well as the strongest predictor of intensive care unit (ICU) admission and in-hospital mortality. Moreover, our data have shown EASIX as a marker of cardiac complications (including arrhythmia and/or acute heart failure), bacterial co-infection and sepsis. EASIX was above the cutoff value of 2.07 in 73 patients who developed acute renal failure, which constituted 83% of patients with this complication. The area under the ROC curve (AUC) was 0.797 (95% Cl: 0.749–0.845) with 64.2% specificity and 83% sensitivity (Figure 1). In the subgroup analysis, EASIX was above the cutoff value of 1.82 in 25 (100%) hematological cancer patients and above the cutoff value of 2.96 in 42 (67%) non-hematological patients with acute renal failure, with AUC of 0.734 (95% Cl: 0.641–0.828) and 0.813 (95% Cl: 0.754–0.872), respectively (Figure 1). Specificity and sensitivity in the hematological cancer subgroup were 45.5% and 100%, respectively, whereas in the non-hematological subgroup, specificity and sensitivity were 77.1% and 83.3%, respectively (Figure 1). 

**Figure 2:**
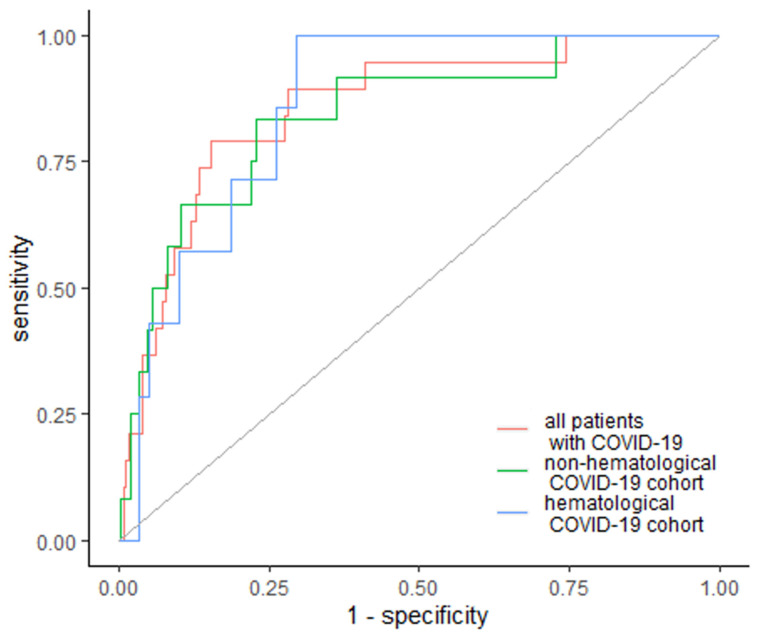
EASIX as a predictor of renal dialysis. Diagonal grey line represents random classifier (false positive rate = true positive rate).

Regarding the need for renal dialysis, EASIX was elevated in 15 (78.9%) patients who required dialysis, including 7 (100%) hematological and 10 (83%) non-hematological patients, with a cutoff value of 4.85 for all patients, 5.51 for the hematological subgroup and 2.96 for the non-hematological subgroup, with an AUC of 0.857 (95% Cl: 0.773–0.940); 0.862 (95% Cl: 0.768–0.956); 0.841 (95% Cl: 0.721–0.961), respectively (Figure 2). Specificity and sensitivity for the need for dialysis were 84.7% and 78.9% for the whole group, 70.3% and 100% for hematological cancer patients and 77.1% and 83.3% for non-hematological patients (Figure 2). 

**Figure 3:**
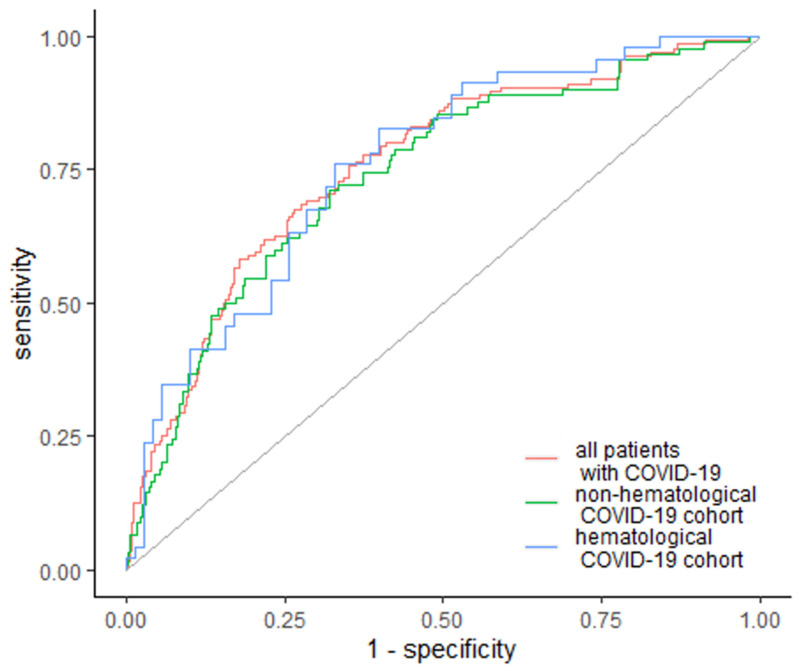
EASIX as a predictor of in-hospital mortality. Diagonal grey line represents random classifier (false positive rate = true positive rate).

EASIX was also the strongest predictor of in-hospital mortality, with higher values in non-surviving patients, with an AUC of 0.759 (95% Cl: 0.711–0.806) and 73.4% specificity and 67.6% sensitivity across the whole studied cohort (Figure 3). The cutoff value was 2.47 in 92 patients (67.6%). Furthermore, EASIX was elevated among subgroups, with a cutoff of 3.16 with AUC of 0.759 (95% Cl: 0.711–0.806) in hematological cancer patients, and a cutoff above 1.96 with AUC 0.74 (95% Cl: 0.682–0.799) in non-hematological non-surviving patients (Figure 3). Specificity and sensitivity were similar for both subgroups: 67.1% and 76.1% for hematological patients, and 68% and 71.1% for non-hematological patients (Figure 3). 

**Figure 4:**
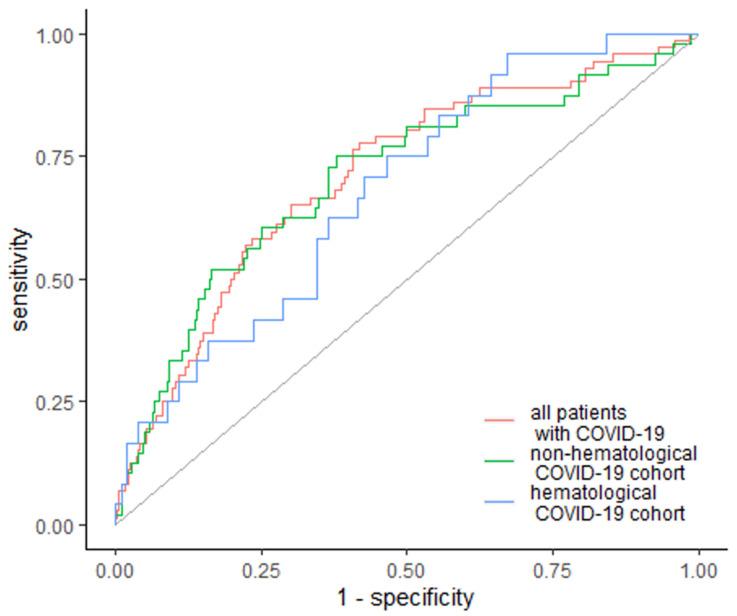
EASIX as a predictor of ICU admission. Diagonal grey line represents random classifier (false positive rate = true positive rate).

Similarly, EASIX was the strongest predictor of ICU admission, with a cutoff value of 1.92 and AUC of 0.711 (95% Cl: 0.645–0.776). The specificity was 58.2%, whereas sensitivity was 77.8% (Figure 4). In the subgroup analysis, EASIX was above the cutoff value of 1.92 and AUC 0.706 (95% Cl: 0.621–0.791) in non-hematological patients, with a specificity of 62% and sensitivity of 75%. In the case of hematological cancer patients, EASIX was above the cutoff value of 1.32 and AUC 0.68 (95% Cl: 0.568–0.792), with a specificity of 33% and sensitivity of 96% (Figure 4). 

EASIX was also a predictor of sepsis occurrence among all patients. The optimal cutoff values of EASIX in these groups of patients were 4.16 and 4.41, respectively, with specificity of 80.8% and 66.4%, and sensitivity of 52.4% and 72.2%, respectively. EASIX values were elevated in 22 (53%) patients who developed sepsis, including 13 (72%) patients with hematological cancer, with an AUC of 0.662 (95% Cl: 0.562–0.762) and 0.680 (95% Cl: 0.545–0.814), respectively.

### 3.4. EASIX as a Predictor of Overall Survival in Hematological and Non-Hematological COVID-19 Patients 

**Figure 5:**
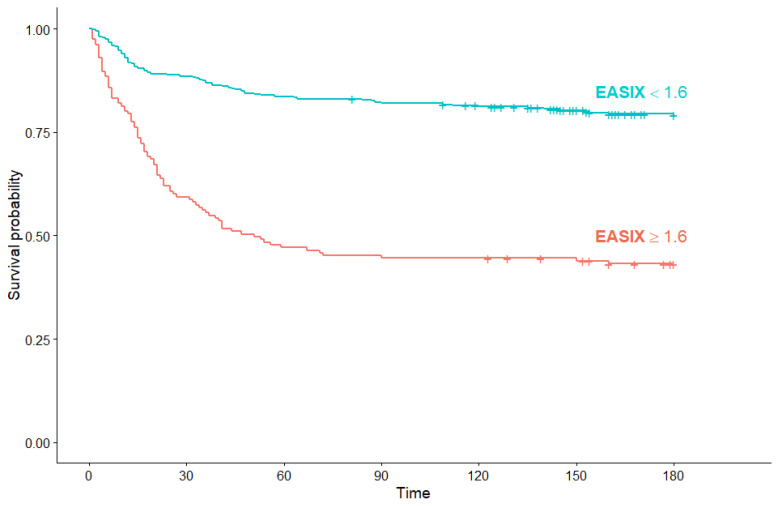
Kaplan–Meier survival curves for overall survival in all COVID-19 patients according to EASIX score.

Across all studied patients, the median OS was not reached. Patients with a high EASIX at diagnosis had a significantly inferior OS compared to the patients with low EASIX (51 days (95% Cl: 34, not reached) vs. median OS not reached) (*p* < 0.001) (Figure 5). 

**Figure 6:**
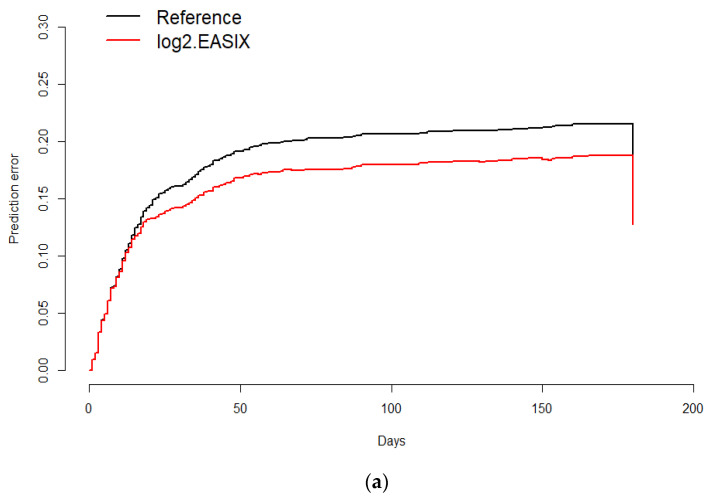

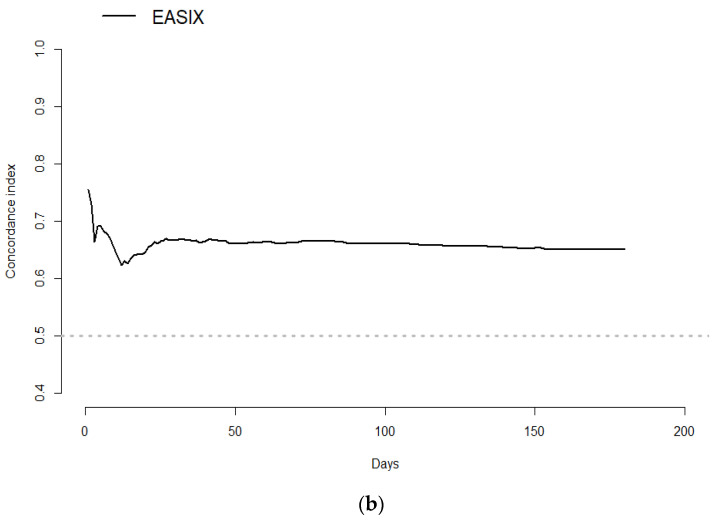
Prediction error curve for overall survival. Prediction error for the validation of the predictive impact of log2EASIX on survival. Black line: reference model (null model). Red line below the reference indicates model validation. Survival (time = 180 days): Reference = 0.187, log2EASIX = 0.16 (**a**); Concordance index for overall survival. Black line: reference model (null model). Survival (time = 180 days): Reference = 0.187, log2EASIX = 0.16 (**b**).

The prognostic value of EASIX for overall survival was validated by calculating the prediction error curve and concordance index curve (Figure 6a,b). A standardized log-rank statistic visualization is shown in Figure S1. A Kaplan–Meier survival curve for overall survival in the validation cohort of 111 hematological patients without COVID-19 according to EASIX score is shown in Figure S2.

In the univariate Cox analysis, the risk of death was increased for high EASIX versus low EASIX (HR 0.274, 95% Cl: 0.202–0.373, *p* < 0.001), the elderly (HR 1.039, 95% Cl: 1.028–1.051, *p* < 0.001), lower hemoglobin (HR 0.815, 95% Cl: 0.769–0.863, *p* < 0.001), higher leukocytes (HR 1.012, 95% Cl: 1.005–1.019, *p* = 0.001) and coexisting coronary artery disease (HR 0.430, 95% Cl: 0.310–0.596, *p* < 0.001). In the multivariable analysis, the risk of death was increased for patients with a high EASIX versus a low EASIX (HR 0.346, 95% Cl: 0.252–0.476, *p* < 0.001) and the elderly (HR 1.034, 95% Cl: 1.021–1.047, *p* < 0.001), and in the case of lower levels of hemoglobin (HR 0.860, 95% Cl: 0.807–0.917, *p* < 0.001) and the coexistence of coronary artery disease (HR 0.653, 95% Cl: 0.454–0.940, *p* = 0.022). Results from the univariate and multivariate Cox analysis are presented in Table 3.

**Figure 7:**
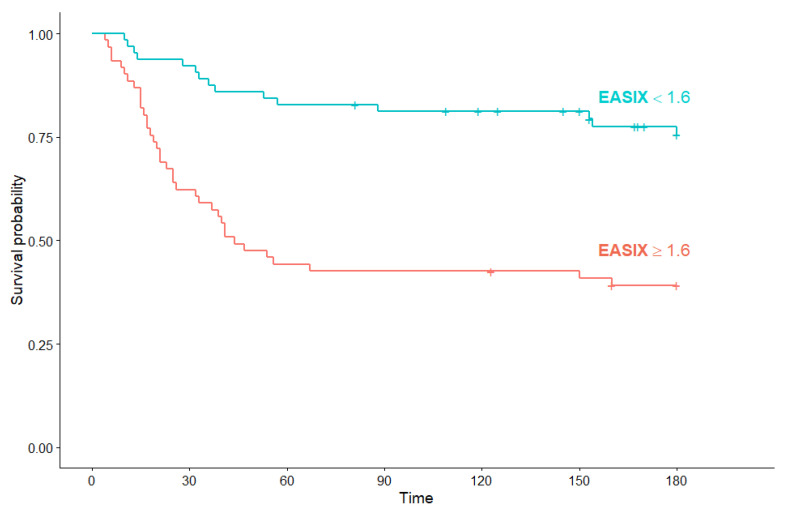
Kaplan–Meier survival curves for overall survival in hematological COVID-19 patients according to EASIX score.

Subgroup analysis for OS regarding EASIX score was performed to determine the prognostic significance of EASIX in hematological and non-hematological COVID-19 patients. In hematological COVID-19 patients with high EASIX, median OS was significantly shorter than in patients with low EASIX (44 days (95% Cl: 32, not reached) versus median OS not reached) (*p* < 0.001) (Figure 7). 

**Figure 8:**
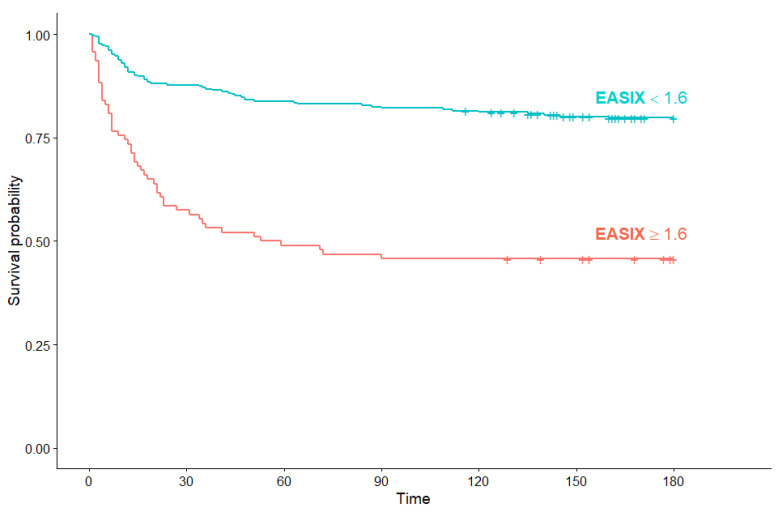
Kaplan–Meier survival curves for overall survival in non-hematological COVID-19 patients according to EASIX score.

Among non-hematological COVID-19 patients, median OS in the high EASIX subgroup was also significantly shorter than in patients with low EASIX (56 days (95% Cl: 23, not reached) versus median OS not reached) (*p* < 0.001) (Figure 8). 

## 4. Discussion

Our study has shown for the first time that EASIX is a universal, early and reliable predictor of the clinical outcome and overall survival in hematological and non-hematological patients diagnosed with COVID-19. The presented study provides three major findings. First, both hematological and non-hematological COVID-19 patients with a high EASIX score at diagnosis showed significantly inferior overall survival compared to patients with a low EASIX score. Second, EASIX is a strong predictor of intensive care unit admission and in-hospital mortality, as well as an indicator of the occurrence of acute renal failure and the need for hemodialysis, both in hematological and non-hematological COVID-19 patients. Third, hematological COVID-19 patients have a higher EASIX score compared to the overall population with COVID-19. Furthermore, in hematological cancer patients compared to non-hematological COVID-19 patients, EASIX is a strong predictor of the occurrence of sepsis during the course of SARS-CoV-2 infection. 

EASIX calculated using LDH, creatinine and platelet levels has been reported as a useful prognostic factor in patients after allogeneic stem cell transplantation, in patients with lower-risk myelodysplastic syndromes and, finally, in newly diagnosed multiple myeloma [5,6,7]. EASIX is an easy tool to predict endothelial dysfunction in the case of hematological disorders. The endothelium is one of the largest organs in the human body and it seems that the pathogenesis of COVID-19 has its source in endothelial activation and inflammation through breaking the vessel barrier’s integrity [13,14,15]. A dysfunctional endothelium in SARS-CoV-2 infection leads to coagulation complications, impaired vascular homeostasis, oxidative stress and, as a result, multiorgan damage [13,14,15,16]. Previous studies have broadly shown the significance of particular components of EASIX in predicting survival in patients with COVID-19. Elevated levels of LDH were related to the risk of developing severe COVID-19 and to increased mortality [8]. Similarly, a meta-analysis of more than 7500 COVID-19 patients showed lower platelet levels in patients with a severe course of disease, as well as in non-survivors [9,10]. Furthermore, COVID-19 might be a reason for acute kidney injury through various mechanisms such as cytokine release syndrome (CRS), which causes systemic endothelial impairment and intrarenal inflammation [11]. Patients who developed AKI due to COVID-19, despite the low prevalence of this complication during the course of disease, are characterized by an especially high mortality rate of 94%; therefore, kidney function should be carefully monitored, especially at the beginning of SARS-CoV-2 infection, to select patients at high risk of the occurrence of acute renal impairment [11]. To the best of our knowledge, our study is the first one assessing the impact of EASIX as a biomarker of endothelial dysfunction on the survival outcome in COVID-19 patients including hematological patients. In our analysis, patients with a higher EASIX had less favorable clinical characteristics, with, more often, dyspnea as a lead symptom of COVID-19, more frequent complications, such as the occurrence of DIC, bacterial co-infection, sepsis, cardiological complications and acute renal failure, as well as a more frequent critical course of COVID-19.

Our study demonstrated a higher EASIX score in hematological compared to non-hematological patients, which indicates more advanced endothelial impairment in COVID-19 patients with concomitant hematological cancer. Across the hematological subgroup of patients, EASIX was a strong marker of sepsis occurrence. It is well known that endothelial dysfunction plays a major role in the development of sepsis and might contribute to increased mortality [17]. Our results showed significantly higher mortality rates in hematological cancer patients with COVID-19 and it has been suggested that, at least partially, adverse outcomes in this subgroup might be caused by doubled endothelial impairment—on one hand, due to underlying malignancy, and on the other, due to SARS-CoV-2 infection.

Across all analyzed patients, we have revealed EASIX as a strong predictor of ICU admission, in-hospital mortality and acute renal failure, as well as the need for renal dialysis. Laboratory parameters of EASIX are the markers of endothelial pathology, which is associated with many processes, such as inflammation and oxidative stress, leading to multiorgan damage and thus the need for intensive treatment. 

Our results show that EASIX might serve as an indicator of ICU admission in all COVID-19 patients. ICU admissions in the case of COVID-19 patients are caused by multiorgan damage, including lung injury and respiratory failure, as well as acute renal failure—all due to endothelial pathology. Respiratory endotheliopathy is closely linked to ARDS by limiting the antithrombotic properties of the endothelium as well as involving alveolar damage, and it seems to be a key feature of critical COVID-19 associated with ICU admission and poor outcomes [18,19]. Similarly, it has been suggested that high creatinine levels resulting in kidney injury and the need for dialysis arise from endothelial damage due to diminished endothelium-dependent vasodilatation [20] and, presumably, in the case of COVID-19, might be a reason for ICU admission and increased mortality. Taken together, the early evaluation of endothelial function calculated by EASIX on admission might be valuable in predicting key organ damage in the course of COVID-19 and the consequent need for intensive treatment.

In a multivariate analysis, we showed an increased risk of death in the case of high EASIX, older age, lower hemoglobin levels and the coexistence of coronary artery disease among all studied patients. Higher EASIX as the expression of significant endothelial damage is associated with increased mortality in all COVID-19 patients. This is especially important considering the fact that the endothelium is one of the main targets of SARS-CoV-2 [21,22] and its dysfunction is responsible for the damage of key organs. Indeed, as we have shown, the COVID-19 course was worse in patients with coronary artery disease as a comorbidity, which involves endothelial impairment. The increased mortality in patients with COVID-19 in the case of anemia, shown in our analysis, might be related to impaired NO bioavailability, which causes endothelial dysfunction as well as vascular organ complications [23].

The presented results demonstrate that COVID-19 patients with higher EASIX on diagnosis had inferior OS compared to patients with lower EASIX, regardless of coexisting hematological malignancy. Therefore, EASIX might serve as a useful early predictor of survival in each analyzed group of patients with COVID-19, including those with concomitant hematological malignancy.

Our study has some limitations. First of all, our analysis is retrospective, and developing a more accurate EASIX prognostic model for COVID-19 patients requires prospective studies, which we will commence in the near future. Secondly, we assessed EASIX at the beginning of SARS-CoV-2, without further evaluation during the course of COVID-19 and the subsequent deterioration in patients’ condition; however, our intention was to determine whether EASIX could stratify prognosis on admission to hospital after SARS-CoV-2 confirmation for better assessment in patients at high risk of an adverse outcome. 

## 5. Conclusions

This study has shown for the first time the prognostic significance of EASIX as a biomarker of endothelial dysfunction in patients with COVID-19, including patients with coexisting hematological cancer. EASIX was a strong predictor of admission to intensive care units, in-hospital mortality and acute kidney failure in all studied COVID-19 patients. COVID-19 patients with high EASIX showed significantly inferior survival outcomes compared to patients with low EASIX, both in hematological and non-hematological subgroup analysis. In addition, hematological cancer patients with COVID-19 had higher EASIX after COVID-19 diagnosis and higher risk of sepsis occurrence in the course of SARS-CoV-2 infection.

## Figures and Tables

**Table 1:** Clinical characteristics of patients with COVID-19 (hematological versus non-hematological).

Variable	All Patients with COVID-19, *n* = 523	Hematological Cancer Patients with COVID-19, *n* = 125	Non-Hematological Patients with COVID-19, *n* = 398	*p*
Age (years)	65 (54–75)	62 (48–70)	66 (56–77)	<0.001
Male, *n* (%)	284 (54%)	80 (64%)	204 (51%)	0.013
Hematological cancer Acute Leukemia/MDS EB2, *n* (%) CLL/indolent lymphoma, *n* (%) High-grade lymphoma, *n* (%) Multiple myeloma, *n* (%) Other, *n* (%)		49 (39%) 27 (22%) 23 (18%) 19 (15%) 7 (6%)		
Comorbidities Hypertension, *n* (%) Diabetes mellitus, *n* (%) Coronary artery disease, *n* (%) Chronic heart failure, *n* (%) Supraventricular arrhythmia, *n* (%) Chronic kidney disease, *n* (%) COPD/asthma, *n* (%)	241 (46%) 85 (16%) 109 (21%) 57 (11%) 50 (10%) 36 (7%) 25 (5%)	60 (48%) 25 (20%) 11 (9%) 50 (13%) 14 (11%) 17 (14%) 4 (3%)	181 (46%) 60 (15%) 98 (25%) 7 (6%) 36 (9%) 19 (5%) 21 (5%)	0.622 0.194 <0.001 0.029 0.459 0.001 0.343
Symptoms/signs of SARS-CoV-2 infection Cough, *n* (%) Dyspnea, *n* (%) Fever, *n* (%) Arthralgia/myalgia, *n* (%)	301 (58%) 294 (56%) 342 (66%) 140 (27%)	76 (61%) 60 (48%) 71 (57%) 40 (32%)	225 (57%) 234 (59%) 271 (68%) 100 (25%)	0.417 0.041 0.019 0.13
COVID-19 complications DIC, *n* (%) Bacterial coinfection, *n* (%) Sepsis, *n* (%) Cardiological complications, *n* (%) Pulmonary embolism, *n* (%) Acute kidney failure, *n* (%) Hemodialysis, *n* (%)	11 (2%) 131 (25%) 42 (8%) 463 (89%) 19 (4%) 88 (17%) 19 (4%)	5 (4%) 35 (29%) 18 (14%) 106 (86%) 5 (4%) 25 (20%) 7 (6%)	6 (2%) 96 (24%) 24 (6%) 357 (90%) 14 (4%) 63 (16%) 12 (3%)	0.142 0.334 0.003 0.429 0.785 0.261 0.179
White blood cells (G/L) Lymphocytes (G/L) Neutrophils (G/L) Basophils (G/L) Eosinophils (G/L) Monocytes (G/L)	6.49 (4.44–8.80) 1.10 (0.70–1.60) 4.20 (2.60–6.43) 0.01 (0.01–0.03) 0.01 (0.00–0.06) 0.50 (0.30–0.70)	6.55 (2.20–9.99) 1.10 (0.3.15) 2.37 (0.69–5.62) 0.02 (0.00–0.10) 0.01 (0.00–0.10) 0.34 (0.10–0.85)	6.46 (4.73- 8.65) 1.10 (0.70–1.50) 4.51 (3.00–6.60) 0.01 (0.01–0.02) 0.01 (0.00–0.04) 0.50 (0.30–0.70)	0.061 0.515 <0.001 0.037 0.027 0.009
Platelets (G/L)	186 (126–253)	95 (41–165)	199 (156–262)	<0.001
Creatinine (mg/dl)	0.91 (0.73–1.19)	0.87 (0.71–1.20)	0.93 (0.74–1.19)	0.182
LDH (U/L)	304 (234–431)	252 (184–376)	327 (251–443)	<0.001
Coagulation INR Fibrinogen (mg/l) D-dimer (ng/mL)	1.11 (1.01–1.26) 3.87 (2.65–5.39) 1.10 (0.59–2.33)	1.15 (1.02–1.35) 3.16 (2.20–4.56) 1.41 (0.76–2.83)	1.10 (1.01–1.24) 4.95 (3.93–6.03) 1.05 (0.58–2.20)	0.026 <0.001 0.073
EASIX score (median log2 EASIX)	0.81 (0.05–1.81)	1.48 (0.36–2.98)	0.68 (0.07–1.55)	<0.001
COVID-19 severity 0—Asymptomatic, *n* (%) 1—Mild, *n* (%) 2—Moderate, *n* (%) 3—Severe, *n* (%) 4—Critical, *n* (%)	28 (5%) 53 (10%) 129 (25%) 240 (46%) 72 (14%)	13 (10%) 23 (18%) 23 (18%) 43 (34%) 23 (18%)	15 (4%) 30 (8%) 106 (27%) 197 (50%) 49 (12%)	0.006 0.001 0.062 0.003 0.089
Time of hospitalization, days	12 (5–18)	11 (1–20)	12 (9–16)	0.094
Clinical outcome, death, *n* (%)	136 (27%)	46 (40%)	90 (23%)	<0.001
COVID-19 specific treatment Oxygen therapy, *n* (%) High-flow nasal oxygen, *n* (%) Mechanical ventilation, *n* (%) Hydroxychloroquine, *n* (%) Lopinavir/Ritonavir, *n* (%) Remdesivir, *n* (%) Convalescent plasma, *n* (%) Tocilizumab, *n* (%) Dexamethasone, *n* (%) Ribavirin, *n* (%) Calcifediol, *n* (%) No treatment, *n* (%)	356 (68%) 74 (14%) 74 (14%) 142 (27%) 54 (10%) 67 (13%) 133 (26%) 13 (3%) 150 (29%) 23 (4%) 12 (2%) 146 (28%)	66 (53%) 36 (30%) 24 (19%) 24 (19%) 2 (2%) 17 (14%) 68 (55%) 4 (3%) 13 (11%) 0 (0%) 0 (0%) 21 (17%)	290 (73%) 38 (10%) 50 (13%) 118 (30%) 52 (13%) 50 (13%) 65 (16%) 9 (2%) 137 (34%) 23 (6%) 12 (3%) 125 (31%)	<0.001 <0.001 0.065 0.025 <0.001 0.739 <0.001 0.519 <0.001 0.006 0.079 0.002

**Table 2:** Clinical characteristics of COVID-19 patients with high EASIX versus low EASIX.

Variable	All Patients *n* = 523	High EASIX (log2EASIX ≥ 1.60), *n* = 155	Low EASIX (log2EASIX < 1.60), *n* = 367	*p*
Age (years)	65 (54–75)	67 (57–77)	64 (53–75)	0.073
Male, *n* (%)	284 (54%)	98 (63%)	186 (51%)	0.009
Comorbidities Hypertension, *n* (%) Diabetes mellitus, *n* (%) Coronary artery disease, *n* (%) Chronic heart failure, *n* (%) Supraventricular arrhythmia, *n* (%) Chronic kidney disease, *n* (%) COPD/asthma, *n* (%)	241 (46%) 85 (16%) 109 (21%) 57 (11%) 50 (10%) 36 (7%) 25 (5%)	87 (56%) 31 (20%) 36 (23%) 24 (15%) 20 (13%) 20 (13%) 6 (4%)	154 (42%) 54 (15%) 73 (20%) 33 (9%) 30 (8%) 16 (4%) 19 (5%)	0.003 0.135 0.392 0.030 0.095 <0.001 0.523
Symptoms/signs Cough, *n* (%) Dyspnea, *n* (%) Fever, *n* (%) Arthralgia/myalgia, *n* (%) Loss of smell, *n* (%)	300 (58%) 293 (56%) 341 (65%) 140 (27%) 84 (16)	79 (51%) 101 (66%) 98 (63%) 39 (25%) 26 (17%)	221 (60%) 192 (52%) 243 (66%) 101 (28%) 58 (16%)	0.047 0.005 0.487 0.578 0.291
Complications DIC, *n* (%) Bacterial coinfection, *n* (%) Sepsis, *n* (%) Cardiological complications, *n* (%) Pulmonary embolism, *n* (%) Acute kidney failure, *n* (%) Hemodialysis, *n* (%)	11 (2%) 131 (25%) 42 (8%) 58 (11%) 19 (4%) 88 (17%) 19 (4%)	7 (5%) 58 (38%) 23 (15%) 30 (19%) 9 (6%) 58 (38%) 16 (10%)	4 (1%) 73 (20%) 19 (5%) 28 (8%) 10 (3%) 30 (8%) 3 (1%)	0.012 <0.001 <0.001 0.001 0.080 <0.001 <0.001
White blood cells (G/L) Lymphocytes (G/L) Neutrophils (G/L) Basophils (G/L) Eosinophils (G/L) Monocytes (G/L)	6.49 (4.44–8.80) 1.10 (0.70–1.60) 4.20 (2.60–6.43) 0.01 (0.01–0.03) 0.01 (0.00–0.06) 0.50 (0.30–0.70)	5.54 (3.09–8.63) 0.79 (0.40–1.20) 3.55 (1.37–6.70) 0.01 (0.00–0.03) 0.00 (0.00–0.02) 0.30 (0.19–0.65)	6.80 (4.83–8.80) 1.20 (0.89–1.80) 4.40 (2.90–6.40) 0.01 (0.01–0.03) 0.01 (0.00–0.07) 0.50 (0.34–0.70)	0.001 <0.001 0.002 0.004 <0.001 <0.001
Platelets (G/L)	186 (126–253)	110 (42–166)	208 (163–288)	<0.001
Coagulation INR Fibrinogen (mg/l) D-dimer (ng/mL)	1.11 (1.01–1.26) 3.87 (2.65–5.39) 1.10 (0.59–2.33)	1.20 (1.08–1.37) 4.19 (2.38–5.65) 1.93 (0.98–3.47)	1.09 (1.00–1.22) 3.74 (2.73–4.99) 0.91 (0.49–1.76)	<0.001 0.768 <0.001
COVID-19 severity 0—Asymptomatic, *n* (%) 1—Mild, *n* (%) 2—Moderate, *n* (%) 3—Severe, *n* (%) 4—Critical, *n* (%)	28 (5%) 53 (10%) 129 (25%) 240 (46%) 72 (14%)	3 (2%) 11 (7%) 32 (21%) 65 (42%) 44 (28%)	25 (7%) 42 (11%) 97 (27%) 174 (48%) 28 (8%)	0.034 0.137 0.163 0.252 <0.001
Time of hospitalization, days	12 (5–18)	13 (5–21)	12 (5–17)	0.380
Clinical outcome, death, *n* (%)	136 (27%)	80 (52%)	56 (16%)	<0.001
Treatment Oxygen therapy, *n* (%) High-flow nasal oxygen, *n* (%) Mechanical ventilation, *n* (%) Hydroxychloroquine, *n* (%) Lopinavir/Ritonavir, *n* (%) Remdesivir, *n* (%) Convalescent plasma, *n* (%) Tocilizumab, *n* (%) Dexamethasone, *n* (%) Ribavirin, *n* (%) Calcifediol, *n* (%) No treatment, *n* (%)	355 (68%) 74 (14%) 74 (14%) 141 (27%) 53 (10%) 67 (13%) 133 (26%) 13 (3%) 150 (29%) 23 (4%) 12 (2%) 146 (28%)	121 (78%) 42 (27%) 45 (29%) 33 (21%) 17 (11%) 24 (16%) 53 (34%) 6 (4%) 48 (31%) 9 (6%) 2 (1%) 39 (25%)	234 (64%) 32 (9%) 29 (8%) 108 (29%) 36 (10%) 43 (12%) 80 (22%) 7 (2%) 102 (28%) 14 (4%) 10 (3%) 107 (29%)	0.001 <0.001 <0.001 0.061 0.655 0.229 0.003 0.184 0.437 0.296 0.327 0.374

**Table 3:** Univariate and multivariate Cox analysis for overall survival in all COVID-19 patients.

Factor	Univariate			Multivariate		
	HR	95% CI	*p*	HR	95% CI	*p*
Male	1.333	0.975–1.823	0.072	1.310	0.949–1.807	0.100
Age, years	1.039	1.028–1.051	<0.001	1.034	1.021–1.047	<0.001
Hb, g/dl	0.815	0.769–0.863	<0.001	0.860	0.807–0.917	<0.001
Log2 EASIX ≤ 1.6	0.274	0.202–0.373	<0.001	0.346	0.252–0.476	<0.001
No CAD	0.430	0.310–0.596	<0.001	0.653	0.454–0.940	0.022

## Data Availability

The data are available from the corresponding author.

## Supplementary Materials

The following are available online at https://www.mdpi.com/article/10.3390/jcm10194373/s1, Figure S1: Cut-point selection for log2 EASIX in the COVID-19 patients, maximal selected log rank statistics, Figure S2: Kaplan–Meier survival curves for overall survival in validation cohort of 111 hematological patients without COVID-19 according to EASIX score.
