# Supplementary material for: Endothelial Activation and Stress Index (EASIX) as an Early Predictor for Mortality and Overall Survival in Hematological and Non-Hematological Patients with COVID-19: Multicenter Cohort Study
Source: J Clin Med. 2021 Sep 24;10(19):4373. doi: 10.3390/jcm10194373 (PMC8509351; doi:10.3390/jcm10194373)
Supplement: Supplementary file 1 [file jcm-10-04373-s001.zip › jcm-1373511-supplementary.pdf]

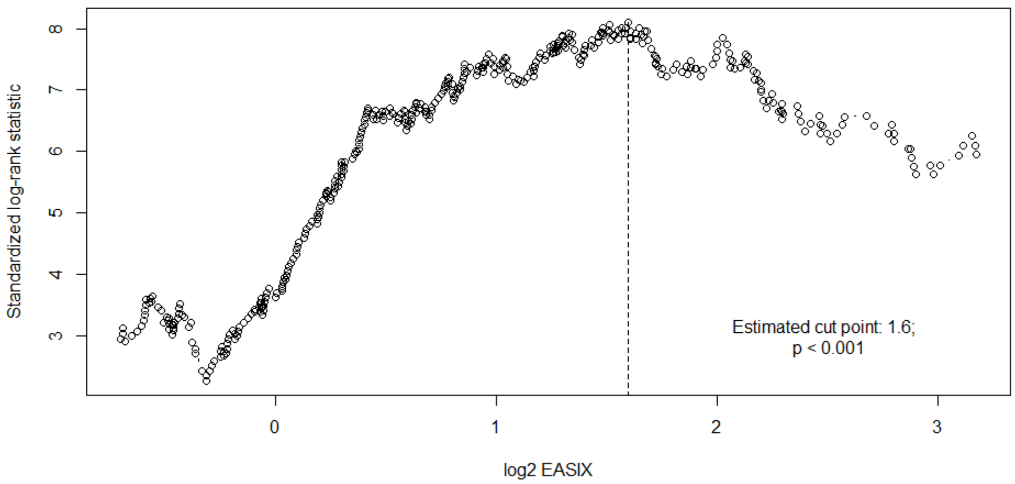

Figure S1. Cut-point selection for log2 EASIX in the COVID-19 patients, maximal selected log rank statistics.

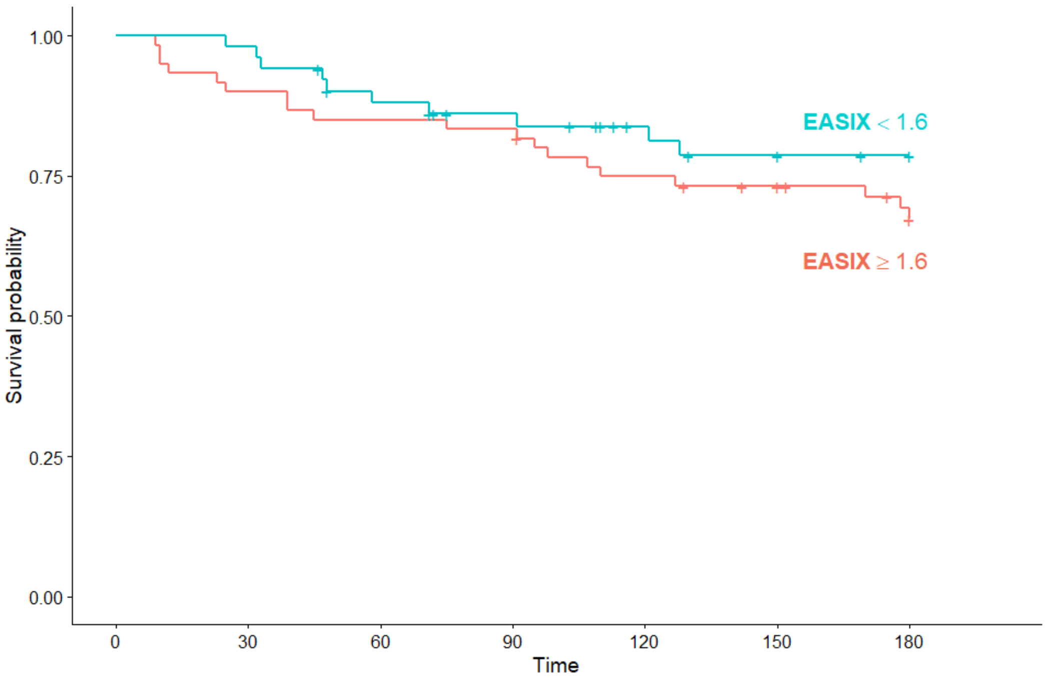

Figure S2. Kaplan-Meier survival curves for overall survival in validation cohort of 111 hematological patients without COVID-19 according to EASIX score.
